# Relationships Between Body Image and Body Parameters in Men Under Long-Term Fasting Conditions

**DOI:** 10.3390/nu17061023

**Published:** 2025-03-14

**Authors:** Alicja Głębocka, Wiesław Pilis, Alicja Żak, Anna Pilis, Karol Pilis

**Affiliations:** 1 Institute of Health Sciences, University of Opole, 45-061 Opole, Poland; aostro@uni.opole.pl; 2 Collegium Medicum, Jan Dlugosz University in Czestochowa, 42-200 Czestochowa, Poland; w.pilis@ujd.edu.pl (W.P.); a.pilis@ujd.edu.pl (A.P.); 3 Department of Economy in Opole, WSB Merito University in Wroclaw, 53-609 Wrocław, Poland; alicja.zak-lykus@opole.merito.pl

**Keywords:** fasting, body image, men, psychological variables, physiological

## Abstract

**Background/Objectives**: Fasting and physical exertion positively affect body mass and composition. This study aimed to examine the effects of physical changes induced by eight days of water-only fasting and a physical exertion test and their influence on body satisfaction and body image parameters in middle-aged men. **Methods**: Fifteen participants were assessed for physical (height, body mass, and body composition) and psychological (body satisfaction, evaluation of body parts, and body image determinants) aspects before and after the fasting intervention, both at rest and post-exercise. Correlation and concordance coefficients were calculated for the analyzed variables. **Results**: It was found that the fasting intervention led to a reduction in body mass and a favorable shift in body composition, while also increasing satisfaction with one’s body and its specific parts. This positively impacted volunteers’ self-assessment of their health status. Selected body image parameters remained at an average level for the male population and did not change following the fasting. Attitudes toward body weight control methods and their perceived effectiveness did not change either. Participants favored reducing food intake and eliminating high-calorie products from their diet, rejecting the use pathological methods typically associated with eating disorders. A few associations between physical and psychological aspects of corporeality were observed, whereas significant correlations between satisfaction with body parts and parameters and a positive body image were confirmed. **Conclusions**: The above suggest that physical conditions and their changes resulting from fasting and physical exertion lead to a positive influence on body satisfaction and its components but do not alter body image.

## 1. Introduction

Body image is a multidimensional structure that can be defined [1,2] as an attitude toward the body that involves emotional, cognitive, and behavioral components, with an emphasis on its external appearance [3]. It can also be considered as the mental image of one’s body, which is formed as a result of an individual’s experiences and interactions with one’s social environment [4]. Therefore, body image can be viewed as a broad category encompassing various aspects of physical appearance, such as satisfaction with one’s body, evaluation of physical attractiveness, and/or body image distortion [5]. As an important structure of the Self, it remains a relatively permanent construct that can be comparatively independent of objective changes in body appearance—both caused by weight gain or loss and those resulting from aging. Given that body image has both state and trait properties, it can exhibit constancy, but it can also change under different circumstances over relatively short periods of time [6].

To reconcile the different ways of understanding, body image can be defined as a system of beliefs and self-evaluations about one’s appearance (cognitive aspect), shaped by internalized attractiveness norms and social information (social aspect) and accompanied by certain emotions (affective aspect) and behaviors (behavioral aspect) [7].

### 1.1. Specificity of Body Image in Men

The factors that are significant in creating body image can be divided into physical, interpersonal, and cultural factors. Physical factors are reflected in personal opinions and feelings about body shape and weight. Interpersonal factors relate to the opinions of those around, especially close ones, and is derived from personal observations and experiences. Cultural factors are linked to ideals of physical attractiveness in specific communities. For instance, in Western culture, slim and muscular figures are promoted as the most desirable, while also conducive to health [8,9]. As a result, depending on the accumulated experience, it is possible to produce a positive or negative form of it. Negative body image is influenced by negative mood and lack of self-confidence [3]. Self-esteem and subjective perceptions of health have been identified as psychological factors influencing body evaluation [10]. The way of building this structure of the Self proceeds differently in men and women. Women are more susceptible to the factors that shape a negative body image, and they remain particularly sensitive to the critical opinions of those around them about their appearance. However, men also respond with a decrease in satisfaction with their body image in response to unfavorable comments from those around them or comparisons with beauty ideals. An important mediating variable between criticism and appearance dissatisfaction is low global self-esteem, which determines how critical messages are interpreted [11,12]. Differences regarding men and women’s body image are seen in the importance of being thin and the level of acceptance of one’s body—both variables are more important for women [13]. In contrast, for male body image, tall height and a muscular physique play an important role. The bodily self also does not play as important a role in the structure of the Self in men as it does in women. Men are less likely than women to form critical opinions about their own bodies and experience fewer negative emotions about their own appearances [11]. They also do not try as hard to correct beauty deficiencies and shortcomings and internalize the prevailing beauty standards to a lesser extent [12].

### 1.2. Body Image and the Use of Fasting

One of the biggest problems facing rich societies today is obesity. The number of overweight people is steadily increasing, contributing to the emergence of psychological, social, and medical problems [14]. People who try unsuccessfully to reduce their body weight often opt for more radical measures. Intermittent fasting is one such weight-loss strategy that can have image and health benefits [15,16,17]. Successful weight reduction can affect body image [11]. On the other hand, erroneous self-perceptions of body weight can limit or nullify the effectiveness of efforts to prevent or reduce obesity [13,17]. Inadequate body image and its role in sustaining well-being are in many cases inextricably linked to obesity [18]. Short-term fasting can exacerbate negative emotions such as anxiety, irritability, or fatigue, and men who highly value the muscularity of the body may feel weaker and less muscular after fasting [19]. For some people, there are positive consequences of fasting—improved mood and an enhanced sense of control [20]. Fasting improves physical, mental, and social quality of life; increases overall vitality; and reduces feelings of fatigue [21] Some studies do not confirm differences between fasting and non-fasting days or between fasting and non-fasting people in terms of experiencing positive and negative emotions [22,23]. Above all, however, intermittent fasting, as an effective strategy for short-term weight loss, contributes to increased satisfaction with body shape and facial appearance and increases self-esteem in terms of the bodily Self [10]. Previous scientific publications confirm the effects of short-term periods of fasting on body image, but there are no reports of studies exploring the importance of long-term fasting.

### 1.3. Somatic Body Image

The somatic image of a person’s body is determined by his height (BH), the size of his mass (BM), fat-free mass (FFM), fat content (BF), total body water (TBW), the sizes of his individual circumferences, and the proportions of the various parts expressed by various weight and height indices. The most popular of these is the body mass index (BMI). The cited data represent the attractiveness of the human body, its degree of self-acceptance, or social acceptance. In addition, this image is supplemented by body posture, the way of moving, facial appearance, and other features. In modern social perception, a slim figure is more accepted and is synonymous with success in life.

The body of an adult men differs significantly from that of a female and is characterized by, among other things, a larger BH, BM, FFM, and TBW and a smaller BF value. In addition, the individual body circumferences of men are larger (excluding relative hip circumference) than those of women. In addition, the male posture is more characterized by strength and decisiveness, and the psychological profile is more firm and rational than that of women.

Body weight can increase significantly or undergo a significant reduction, where its composition changes fundamentally. Increased body weight expressed by a BMI of more than 25 kg/m^2^ is referred to as overweight or obesity and can, in extreme cases, reach up to several hundred kilograms. This is the result of a positive energy balance [24] and/or pathological conditions [25] as well as excessive accumulation of adipose tissue. Such a picture of the human body is not universally accepted. In people who participate in strength sports, body weight is often elevated, and BMI can significantly exceed the limit of 25 kg/m^2^, which is due to increased muscle mass without increased fat. In some members of the public, this finds acceptance or even arouses admiration. At the same time, in the average person, excessive body weight is considered a cause of many diseases; therefore, it is recommended to reduce it.

Weight reduction and a change in body composition can be achieved by eating a different type of diet [26,27,28]. This includes limiting sugars [29] and/or fats [30], changing the frequency of food intake [31], using dietary supplements [32], drinking optimal amounts of water [33], or opting for bariatric surgery [34]. In extreme cases, weight reduction is accomplished using appetite suppressants [35] and laxatives [36] or provoking vomiting [37]. Professional athletes use other, specific methods of reducing body weight [36]. An important way to reduce body weight and changes in body composition is the use of long-term fasting. In our earlier study, 8 days of water-only fasting resulted in reductions of BM by 6.92%, BF by 12.49%, FFM by 5.80%, TBW by 5.64%, and BMI by 7.01% [38]. More drastic changes were registered in a 34-year-old obese man who underwent a 50-day fasting. His body weight during this time was reduced from 96.8 kg to 76.4 kg (21.07%), and his BMI decreased from 30.2 kg/m^2^ to 23.5 kg/m^2^ (22.19%), depicting a normal body composition [39]. A classic experiment conducted in the state of Minnesota (USA) also showed that a person’s malnourished state can affect his cognitive and social functions in addition to somatic changes [40].

The prevalence of overweight and obesity among highly developed societies forces people to reduce excessive body weight, and the use of long-term fasting for this purpose is hardly tolerated by them. Therefore, attempts are being made to reduce body weight by practicing less restrictive forms of fasting [41,42,43,44].

An important role in the reduction of body weight and positive modification of its composition is played by increased physical activity used mainly in the form of endurance and strength training, both by healthy and sick people [45]. Combining exercise and dietary modifications can, in addition to other health-promoting aspects, enhance weight reduction and modification of body composition [46,47,48].

We decided to use an 8-day fasting because different populations of people need to quickly reduce body weight, e.g., some groups of athletes or business people, and such an intervention is effective in this respect. Fasting of this duration is not harmful to health and is relatively easily tolerated. However, it should be noted that people undergoing fasting intervention should rest and drink plenty of water to minimize the degree of dehydration, which would have a negative impact on the appearance of the body.

It should be mentioned that significant weight reduction and change in body composition occurs under conditions such as malnutrition, cachexia, metabolic diseases, mental anorexia, mental bulimia, or many other conditions [40,49]. The extreme low BMI in one of these conditions was 12 kg/m^2^ [50].

With the above data in mind, in the present study, an attempt was made to evaluate selected components of corporeality based on physical and psychological aspects—body image and factors determining it in middle-aged men participating in 8 days of water-only fasting and additionally loaded with maximum intensity exercise.

The following research hypotheses were adopted:

(1) The consequences of long-term fasting will be somatic changes that include the following:
  - weight loss;
  - decrease in BMI;
  - decrease in the proportion of fat in total body weight.
(2) Long-term fasting will be followed by positive changes in the cognitive, emotional, behavioral, and social components of body image.
(3) Positive changes in the physical aspects of corporeality will positively correlate with a positive body image in psychological terms.

## 2. Materials and Methods

### 2.1. Participants

Eighteen candidates were enrolled in the study, including 16 men and two women. During the study, one man and two women chose not to continue fasting.

Eligible for the study were subjects who had previously participated in fasting sessions of at least 7 days’ duration, during which they consumed only mineral water. The subjects were in good psychophysical condition before and during the fasting intervention and tolerated the lack of food intake well. The subjects thought positively and were characterized by strong motivation to participate in the next fasting session in their lives. They expected this fasting intervention to improve their health, just as they had done previously. The volunteers remained in their place of residence during the 8-day of water-only fasting and fulfilled their daily work and family obligations. The group consisted of government officials, businessmen, and academics and passed medical examinations. Three days before the start of the study, during the study, and 14 days after the end of the experiment, the volunteers had telephone contact with the study organizer, doctor, and psychologist. During these periods, no cognitive, emotional, behavioral, or somatic disorders were found in the subjects. Volunteers were familiarized with the methodology and conditions of the experiment. They were also reminded of the safety conditions during and after the fasting intervention. Participation in the study was determined by volunteers meeting the following criteria: (1) previous experience in conducting fasting practices; (2) no use of restrictive diets within the past 6 months; (3) no use of cigarettes, alcohol, or drugs; (4) no acute or chronic mental or somatic illness of any kind; and (5) the absence of eating disorders and mental dysfunctions. The participants were also informed of the possible negative health effects of the experiment and especially the fasting intervention, after which the men gave their written consent to participate in this study.

The research protocol was also approved by the Research Ethics Committee of the Jan Długosz University in Częstochowa (Poland; KE-0/1/2019; 5 March 2019) and was conducted in accordance with the requirements of the Declaration of Helsinki.

### 2.2. Procedure

Male volunteers reported to the laboratory in the morning in the fasting state. At the beginning, the examined men, under the supervision of a psychologist, completed the following tests: the Body Parts and Parameters Satisfaction Scale (BPPSS), the Scale of Weight Control Methods SWCM, the Weight Control Effectiveness Rating Scale (WCERS), and the Body Image Questionnaire (BIQ), all of which are methods reported by A. Glebocka. In addition, the study used the Body Esteem Scale (BES) by S. Franzoi and S. Shields in the form of the Polish adaptation by M. Lipowska and M. Lipowski; the Rosenberg Self-Esteem Scale (RSES) in the form of the Polish adaptation by M. Laguna, K. Lachowicz-Tabaczek, and I. Dzwonkowska; and the Lying Scale (LS) from H. Eysenck’s EPQ-R test in the form of the Polish adaptation by P. Brzozowski and R. Drwal [12,51,52].

In the next step, venous blood was collected from the volunteers for determination of β-hydroxybutyrate (β-HB) and cortisol (C) levels. Then, anthropometric and somatic measurements were taken, and the volunteers were interviewed. In the next stage, the men performed an exercise test on a bicycle ergometer with gradually increasing intensity up to maximum power. The body load of the exercise was recorded based on heart rate. At 3 min after the end of the exercise, venous blood was again drawn, and body weight and body composition were again measured. After these activities, fasting was continued for 8 days, during which only arbitrary amounts of moderately mineralized water were consumed.

After the fasting ended, all measurements taken before its start were repeated.

### 2.3. Measurements

#### 2.3.1. Demographic and Lifestyle Measurements

Finally, 15 men with an average age of 59.8 ± 12.37 years were included in the analyses. Detailed somatic data along with age, diet presentation, and social conditions (obtained during the interview) of the studied men are presented in Table 1.

#### 2.3.2. Anthropometric and Somatic Measurements

Body height (BH) was measured with an anthropometer (A-226, Tristom, Olomouc, Czech Republic). Body weight (BM) and its components, i.e., fat content (BF), fat-free mass (FFM), total water content (TBW), and body mass index (BMI), were determined using a Tanita TBF 300A body composition analyzer (Amsterdam, The Netherlands).

#### 2.3.3. Analysis of Blood Samples

Blood collected from the elbow vein was initially stored at room temperature for no longer than 10 min. In the next step, the blood was centrifuged at 1500× *g* for 15 min, and the obtained serum samples were frozen at −80 °C and stored for further analysis. MAGLUMI (Cortisol-CLIA, Shenzhen New Industries Biomedical Engineering Co., Ltd., Shenzhen, China) assays were used to determine serum cortisol (C) concentrations; reference values for this variable are 52–350 ng/mL. The concentration of β-hydroxybutyrate (β-HB) was determined using the RANBUD diagnostic kit from Randox, Laboratories Ltd., Crumling, UK. Reference values for serum are—0.03–0.3 mmol/L.

#### 2.3.4. Assessment of Psychological Variables

Dimensions related to body image were examined using the following: (a) The Body Parts and Parameters Satisfaction Scale (BPPSS) by A. Głębocka measures on a nine-point Likert scale (1 means very dissatisfied, 9 means very satisfied) satisfaction with nine body-related dimensions, including seven body parts and weight and height (Cronbach’s alpha = 0.86). (b) The Scale of Weight Control Methods (SWCM) by A. Głębocka refers to various weight control methods, such as eliminating sugar/sweets from the diet or taking an appetite suppressant, rated by respondents on a nine-point scale with 1 indicating never used and 9 indicating use regularly (Cronbach’s alpha = 0.81). (c) The Weight Control Effectiveness Rating Scale (WCERS) by A. Głębocka is used by respondents to rate the effectiveness of each method (1 means totally ineffective, 9 means very effective) (Cronbach’s alpha = 0.81). (d) The Body Image Questionnaire (BIQ) consists of 40 statements rated by respondents on a five-point scale, where 1 means completely disagree and 5 means completely agree. The questionnaire contains four subscales: (i) the cognition-emotions subscale is used to measure opinions about one’s own appearance, such as I feel pressure from my immediate environment to lose weight, and the affective component, such as feelings (positive/negative) about one’s body, attitude toward one’s appearance, general mood, and feelings of guilt or anxiety, e.g., I am ashamed that I look like this, or I am worried about my physical appearance (Cronbach’s alpha = 0.93); (ii) the behavior subscale contains statements that relate to a healthy lifestyle such as I like to relax actively (Cronbach’s alpha = 0.83); (iii) the external criticism subscale of the social environment allows us to determine the subjective level of acceptance of the respondent by the environment, which is expressed in critical remarks about his or her appearance (Cronbach’s alpha = 0.76); and (iv) the pretty-ugly stereotype subscale, measures the degree of internalization of contemporary beauty standards, e.g., a slim body is preferred in our culture, and awareness of negative stereotypes of obese people, e.g., obese people have trouble finding a partner (Cronbach’s alpha = 0.88). The higher the score on the scale is, the more negative the body image. The method underwent a validation procedure, which showed its high reliability (Cronbach’s alpha = 0.93) and accuracy [12]. (e) The Body Esteem Scale (BES) by S. Franzoi and S. Shields in the form of the Polish adaptation by M. Lipowska and M. Lipowski examines people’s feelings toward their own corporeality. In the version for men, the scale has 3 subscales: Physical Attractiveness (Cronbach’s alpha = 0.85), Body Strength (Cronbach’s alpha = 0.85), and Physical Conditioning (Cronbach’s alpha = 0.88). The higher the score on the scale is, the more positive feelings toward one’s own body the respondent has [53]. (f) The Rosenberg Self-Esteem Scale (RSES) in the Polish adaptation by M. Laguna, K. Lachowicz-Tabaczek, and I. Dzwonkowska measures global self-esteem treated as a fixed trait (Cronbach’s alpha = 0.81) [51]. The EPQ-R Lie Scale by H. Eysenck in the form of the Polish adaptation by P. Brzozowski and R. Drwal measures the need for social approval and, thus, the tendency to present oneself in a more favorable light (Cronbach’s alpha = 0.78) [52].

### 2.4. Statistical Analyses

The data obtained in the study were presented as arithmetic means (Ms) and standard deviations (±SDs). Normality was measured using the Shapiro–Wilk test. Differences between pre- and post-fasting measurements were analyzed using the *t*-test for paired data and the Wilcoxon paired *t*-test. Cohen’s d was used to calculate effect size. Standardized effects were defined as small (>0.2), moderate (>0.5), and large (0.8). Differences between dependent variables in the first measurement (before fasting) and variables in the second measurement (after fasting) were examined using the Friedman ANOVA test and Kendall’s concordance coefficient. To determine the effect size in this procedure, partial eta-squared (ηp^2^) was calculated. The effect size was defined as follows: η^2^ = 0.01—small (S); η^2^ = 0.06—medium (M); and η^2^ = 0.014—large (L).

In addition, Spearman’s rank order correlation analyses were performed to determine the relationships between body image indices and physiological factors. Values at *p* < 0.05 were considered statistically significant. Calculations were made using Statistica, version 13.

## 3. Results

The study conducted focused on two areas related to human corporeality—the physical and mental spheres. Of interest were not only the changes in these spheres caused by fasting but also the interrelationships between them.

### 3.1. Comparisons of Pre- and Post-Fasting Measurements

#### 3.1.1. Comparisons of Physiological Indicators

BM and its components described by absolute values were significantly reduced by the fasting intervention. In contrast, serum β-HB and C concentrations significantly increased (*p* < 0.001) both at rest and after exercise during 8 days of water-only fasting. As a percentage of BM, its components, i.e., BF (at rest only), FFM, and TBW, did not change after the fasting intervention. The exercise test used did not change BM and its components or serum β-HB and C concentrations (Table 2). During the study’s exercise test, the men achieved a similar maximum heart rate of 162.2 ± 8.24 bpm before the fasting intervention and 161.8 ± 9.17 bpm after.

The respective values of η^2^ and test power for physiological variables were as follows: BM [kg]—0.076, 0.590; BF [%]—0.004, 0.079; BF [kg]—0.013, 0.134; FFM [%]—0.005, 0.083; FFM [kg]—0.093, 0.663; TBW [%]—0.004, 0.076; TBW [kg]—0.097, 0.691; BMI [kg/m^2^]—0.060, 0.474; β-HB [mmol/L]—0.787, 1.000; and C [ng/mL]—0.466, 0.999.

#### 3.1.2. Comparisons of Body Image Dimensions

Presentations of dimensions related to psychological aspects of corporeality begin with a summary of data on current and desired body weight. In Table 3, the data show that the weight of the subjects decreased statistically significantly, but the preference for the most desirable weight did not change. On average, the weight of the subjects decreased by 6.273 kg and approached the desired weight. The difference between the post-fasting weight and the most desirable weight was not statistically significant. In addition, with the decrease in weight, a significant increase in the subjects’ evaluation of their own health was observed. Detailed data are included in Table 3.

Subsequent measurements referred to the level of satisfaction with the appearance of selected parts and body parameters. Comparisons between data collected before and after fasting show statistically significant differences. They mainly concern those body parts that are most associated with body weight and are most deformed by excessive weight gain, namely the waist and abdomen. In addition, after fasting, the subjects were more satisfied with their chest and arms. Satisfaction with other body parts and parameters did not change after fasting, including satisfaction with body weight. Detailed data are shown in Table 4. The respectively values of Cohen’s d and test power for the variables above were as follows: body weight—4.221, 1.000; desired weight −4.907, 1.000; and health assessment—0.775, 0.535.

The analyses on body satisfaction are worth supplementing with comparisons between variables in measure I and measure II. They show that the respondents were least satisfied with their abdomens and waistlines. This pattern did not change after the fasting. Before the fasting, the subjects were most satisfied with their faces, followed by their arms and height (see Table 5). After the fasting, the source of greatest satisfaction was the shoulders, height, and face. However, it should be noted that after the fasting, basically all indications exceeded the average for the scale, which is five points. Before the fasting, only satisfaction with the waist and abdomen was lower than the average for the scale. This means that respondents generally had high levels of satisfaction with body parts and parameters (see Table 4). The respective values of Cohen’s d and test power for psychological variables were as follows: face—5.422, 1.000; chest—1.549, 0.984; arms—2.840, 1.000; waist—0.516, 0.277; belly—0.645, 0.400; thighs—4.410, 1.000; legs—6.584, 1.000; height—5.809, 1.000; and mass—2.711, 1.000.

Another element that makes up the psychological aspect of corporeality is body image. In this study, it was measured using the BIQ and BES. Comparisons between measurements taken before and after fasting were not statistically significantly different for any variable. This means that the use of fasting neither modifies beliefs about one’s own body and physical attractiveness, nor the emotions felt toward the body. Neither does affect the subjects’ beliefs about how their physical attractiveness is judged by those around them and the degree to which they internalize the stereotypes according to what is physically attractive, i.e., thin, people have more positive attributes and lead better lives than unattractive, i.e., obese people. In addition, the study included the SES, which measures global self-esteem. Here, too, there were no significant differences between measurements before and after fasting. A comparison of the SES results to norms for the Polish male population shows that the subjects had slightly elevated self-esteem (7 sten). Measurements with the Lie Scale (L Scale)-EPQ-R prove that in giving their answers, the subjects were not guided by the need for self-presentation toward better adjustment, so the results obtained in all questionnaire measurements should be considered reliable (5 sten). Detailed data are presented in Table 6.

Interestingly, global self-esteem as measured using SES correlated significantly only with the pretty-ugly stereotype subscale (−0.679) and the body strength subscale (0.60). This means that the higher self-esteem the respondents had, the less they shared stereotypical opinions about obese people, and the higher they rated body strength in themselves, understood as having typically masculine attributes of physique and fitness.

In order to provide a full account of the relationships between the various dimensions of body image, analyses were performed on the differences between the various variables in measure I and measure II. They show that for the BIQ, the pattern of relationships between subscales was identical before and after fasting. The relatively most positive aspect of the subjects’ body image was their belief that those around them evaluate their physical attractiveness positively and that they do not experience criticism from other people in this area. The most negative element of body image was the relatively strong internalization of pretty-ugly stereotypes. However, it should be noted that the internal differences in the averages obtained in the various subscales of the BIQ cannot be taken as an objective indicator of body image of the subjects. This is because when we compare the results obtained by them to the norms for the adult male population, we see that they obtained results in all subscales oscillating within the limits of average results, i.e., from 4 to 6 sten. A similar situation occurred in the BES measurements. In this study, subjects obtained results typical of the normalization population. The differences between the subscales in measurements I and II boiled down to the fact that before fasting, the mean for the Body Strength subscale differed significantly from the mean of the Physical Attractiveness subscale. In measure II, there were no statistically significant differences between the BES subscales. The respective values of Cohen’s d and test power for psychological variables were as follows: cognition-emotions—11.361, 1.000; behaviors—4.131, 1.000; external criticism—4.518, 1.000; pretty-ugly stereotype—9.553, 1.000; physical attractiveness—9.682, 1.000; body strength—8.391, 1.000; physical condition—12.006, 1.000; Self-Esteem Scale—7.617, 1.000; and Lie Scale (L Scale)-EPQ-R—10.070, 1.000. Detailed data are provided in Table 6 and Table 7.

The final aspect of body image that was more extensively covered in the study was that of behavior toward one’s own body relating to eating habits. The respondents answered two questions with regard to the same specific methods of weight control: whether they use a specific method and how they evaluate its effectiveness. In addition to fairly typical and commonly used methods like limiting the amount of food consumed and eliminating certain products, there were also methods that in science and clinical practice are considered pathological forms of weight control like provoking vomiting or taking laxatives. A comparison of the results before and after fasting shows that it did not change the methods of weight control used by the subjects, which seems obvious. Moreover, it did not affect the evaluation of the effectiveness of weight control methods included in the scale (see Table 8).

Analyses of the weight control methods used and the evaluation of their effectiveness yield interesting results when comparing the differences between the various variables that occurred in study I and study II. All of the measurements included in Table 8 differ statistically significantly. Thus, for example, the subjects use food restriction significantly more often than appetite suppressants, laxatives, or provoking vomiting. In the use of specific methods of weight control, a clear line can be seen between those involving limiting the amount of food consumed or eliminating products considered harmful to health and those that are pathological and involve eating disorders. In assessing the effectiveness of weight control methods, respondents considered fasting and limiting the amount of food consumed and eliminating sugar/sweets from the diet to be the most effective. They rated significantly lower the use of restrictive diets and the elimination of meat and fatty foods from the diet. They rated the lowest the effectiveness of pathological forms of weight control, that is, taking appetite suppressants and laxatives and provoking vomiting (see Table 8 and Table 9). The respective values of Cohen’s d and test power for variables presented in Table 8 were as follows: i. using different ways to control body weight: limiting the amount of food—5.746, 1.000; elimination of sugar—3.357, 1.000; eliminating fatty foods—5.293, 1.000; eliminating meat—3.227, 1.000; restrictive diet—3.486, 1.000; fasting—6.584, 1.000; appetite suppressants—0.258, 0.105; use of laxatives—3.098, 1.000; and provoking vomiting—0.000, 0.050; ii. evaluation of the effectiveness of different weight control methods: limiting the amount of food—8.133, 1.000; elimination of sugar—8.018, 1.000; eliminating fatty foods—8.521, 1.000; eliminating meat—7.746, 1.000; restrictive diet—8.004, 1.000; fasting—1.549, 0.984; appetite suppressants—1.291, 0.927; use of laxatives—2.711, 1.000; and provoking vomiting—0.516, 0.277.

### 3.2. Correlation Analyses

Table 10 contains correlations between body composition indices and satisfaction with appearance and body parameters. It turned out that statistically significant correlations occurred here incidentally and only in measurements after fasting. Thus, in resting and post-exercise measurements, satisfaction with body weight correlated negatively with FFM [kg], TBW [kg], and BMI.

Correlations were even less frequent between body composition indices and body image dimensions. In this case, in contrast, correlations appeared in the pre-fasting measurements and were only for BMI, which positively correlated with the cognition-emotions subscale and the BIQ ambient criticism subscale, both at rest and after exercise. The remaining correlations did not reach the level of statistical significance (see Table 11).

Finally, to fully illustrate the relationship between psychological aspects of corporeality, correlations between satisfaction with appearance and body parameters and body image indices are presented. In many cases, there were not only statistically significant, but very strong correlations. Based on the results obtained, it can be generally concluded that body satisfaction in the subjects has a clear relationship with their positive body image. In principle, significant correlations with body image indicators appeared for all body parts and parameters. Given the nature of the research presented here, it is noteworthy that in measurements taken before the fasting, satisfaction with the abdomen significantly correlated with six of the seven body image indicators included in the study and satisfaction with the waist with five. In measurements taken after the fasting, significant correlations appeared for the waist in one case and the abdomen in three.

In addition, it can be said that beliefs about one’s own body and the emotions that this body evokes had a strong relationship with satisfaction with face, chest, waist, abdomen, and height before fasting and face, chest, abdomen, height, and weight after fasting. Behaviors toward one’s own body were related to satisfaction with the chest, waist, and abdomen before fasting and with the chest and thighs after fasting. Feelings of being accepted or criticized by those around them were related to satisfaction with face and height in both measurements and with abdomen before fasting. The degree of internalization of the pretty-ugly stereotype was related to satisfaction with height in both measurements and additionally to satisfaction with face, waist, and abdomen before fasting and abdomen and thighs after fasting.

In contrast, feelings of physical attractiveness correlated significantly before fasting with satisfaction with the chest, arms, and thighs and after fasting with satisfaction with the face and legs. Body strength had a strong relationship before the fasting with satisfaction with the chest, arms, waist, abdomen, and thighs and after with satisfaction with the chest and legs. Finally, before the fasting, the physical condition score was associated with satisfaction with the chest, arms, waist, abdomen, and thighs and after the fasting with satisfaction with the face, waist, abdomen, and legs. Detailed data are shown in Table 12.

## 4. Discussion

The experiment conducted took place under conditions of comfort (study I) and increased stress caused by the fasting intervention (study II). The measure of the intensified stress response was elevated C levels [54], and the expression of a significant metabolic change related to the brain, muscles, and other organs was increased ketosis manifested by elevated serum β-HB levels [55] indicative of the volunteers’ compliance with the fasting conditions.

### 4.1. Somatic Conditions and Body Perception During Fasting and Physical Exercise

Under the conditions of the fasting intervention and physical exercise, as well as the changing BM and its composition, the volunteers subjectively assessed improvements in their health. Perhaps it was these positive somatic changes that caused their good psychological well-being, which we observed in earlier studies [56], and this influenced them to express a positive health opinion [21]. It should be added that the men surveyed struggled with overweight (BMI~27.8 kg/m^2^) caused mainly by excess body fat (~23.5%). At the same time, the volunteers had the knowledge that being overweight or obese is detrimental to the body and can cause many dangerous diseases [14,57,58]. Thus, taking health-promoting measures probably compounded their well-being. Another reason for the study men’s good mental attitude, occurring after the fasting intervention, which may have influenced their opinion of good health, was the fact that they achieved a similar maximum heart rate during the exercise ergometric test that was not statistically different before and after 8 days of water-only fasting. Although the exercise test was performed only twice, lasted a relatively short period, and did not cause significant somatic changes, it should be assumed that it was a source of satisfaction with maintaining a relatively high exercise capacity despite the end of the fasting intervention.

Male respondents expressed a significant level of satisfaction with the changes in the chest, arms, waist, and abdomen caused by 8 days of water-only fasting [19]. These indications seem reasonable especially with regard to the chest, as its appearance indicates a normal posture and satisfactory cardiorespiratory capacity. A deformed chest of the funnel-shaped or chicken-shaped type with humps, depressions, or scoliosis decreases the functional efficiency of the lungs and heart and affects a low opinion of this part of the body as well as a lack of satisfaction with it. It should be added that conservative or surgical treatment of such defects is lengthy, sometimes dangerous to health, and often ineffective [59]. Volunteers’ expressions of satisfaction with the appearance of the waist and abdomen are quite common, since it is excessive waist circumference that most distorts the figure, affects the development of cardiovascular disease, and almost always accompanies obesity [60]. Among the many even technologically very advanced diagnostic tools indicating future or currently developing pathologies of this system is an extremely simple measurement, which is the waist circumference. This circumference above 94 cm in men indicates a significant risk of cardiovascular diseases or certain metabolic diseases [61]. It should also be noted that increased waist circumference is usually associated with the accumulation of excess visceral fat in this place, which is a significant factor in the occurrence of cardiovascular diseases, especially in men [62]. In addition, increased waist circumference is accompanied by a thickened skin and fat fold, which lowers the aesthetic image of this part of the body, mainly in people with excessive weight. Such people often resort to reducing waist circumference and thus fat content using various methods even including life-threatening and health-threatening bariatric surgery [63]. On the contrary, in thin people and mainly in those who are physically active or in professional athletes, the waist is proportionally narrower, the folds of skin and fat are significantly thinner, the amount of abdominal fat tissue is smaller, and the straight and oblique muscles of the abdomen are clearly outlined. This creates an impeccable image of this part of the body with great aesthetic value, ensuring a positive perception of one’s own body [64,65]. In these people, the risk of cardiovascular disease is low.

Invariably, however, BH, face, and arms were the greatest source of satisfaction among the different body parts in the men surveyed both before and after fasting [10]. These facts seem to confirm general observations that tall people enjoy more popularity and are more often presented in the media, especially men. Unfortunately, BH is significantly determined genetically, and its correction using hormonal or surgical therapies can be ineffective or detrimental to health. Significant BH also determines its favorable proportions, which can also be a source of satisfaction and account for a person’s positive social perception and image. A large BH usually entails the presence of a significant length of the lower limbs, which has a positive effect on body proportions and body image in both women and men [65]. Unfortunately, in our study, respondents did not pay significant attention to the appearance of the legs. Facial appearance played a similar important role as BH in public perception and body image and is more important in women than in men [10]. Also, the face largely or very largely determines satisfaction with body acceptance and projects body image, which is related to a person’s media presence or popularity. Therefore, facial appearance is a significant focus of cosmetology and plastic surgery, mainly among women. The indication of the arms as a part of the body that gives satisfaction to the surveyed volunteers may be related to the identification of men as people characterized by considerable strength, which translates into their large circumference and their considerable strength [19]. This is manifested especially by people who train strength sports [12,66] and, together with BH and facial appearance, affects a person’s body image. Our study, however, showed that body image did not change after the applied fasting intervention, although its determinants included such factors as physical attractiveness, body strength, and physical condition [22,23]. These results can be taken as further evidence that body image, as a structure of the Self, is a permanent construct and is unlikely to change under the influence of short-term current experiences [3,4,5,6]. Certainly, the age of the volunteers was important for the stability of body image because people around the age of 50 achieve relative stability of features [67]. Another result of the conducted research, which turned out to be inconsistent with expectations, was the highly selective relationship between somatic and mental parameters of the body and its mental representation. It appears that this mental representation is formed primarily on the basis of experiences other than somatic ones, mainly observations, beliefs, emotions related to the body, and opinions of other people and references to standards of attractiveness [7,12].

### 4.2. Other Methods of Controlling Body Structure and Appearance

In our study, among the most common methods used to control BM both before and after the fasting intervention, volunteers mentioned “physiological” ways, including food restriction, elimination of sugar and fat from the diet, restrictive diets, or fasting interventions. The volunteers considered food restriction and elimination of sugar from the diet and fasting interventions to be the most effective for weight control in both conditions: before and after 8 days of water-only fasting. The aforementioned ways of reducing BM [16] and their effectiveness have been confirmed in the scientific literature [30,36]. It seems that the fasting intervention, although it cannot be maintained for too long, exerts the most spectacular and immediate effects not only in the form of BM reduction and changes in its composition, but also improves urogenital function, economizes endocrine function [38], improves carbohydrate control, activates fat metabolism [68], and leads to better well-being [56]. A positive choice of the subjects was the rejection of so-called “pathological” methods of BM control and their effectiveness in this regard both before and after the fasting intervention. These methods can cause and/or accompany numerous diseases associated with eating disorders [69]. The discussed methods of controlling BM and its composition are used in medical treatment, in intentional BM reduction, or in competitive sports [36]. In addition to the methods of BM control mentioned in our study, there are many others; among them, exercise plays a serious role. It is well known that physical activity plays an important role in the regulation of BM and its composition, when it is present in daily life and characterized by a longer duration and a correspondingly high intensity [70].

In cases of significant obesity or morbid obesity, however, it is necessary to simultaneously include pharmacological therapy in the form of drugs: modifying absorption in the gastrointestinal tract (orlistat), acting on the central nervous system by regulating appetite (phentermine-topiramate and naltrexone-bupropion), or hormonal drugs stimulated by nutrients in the gastrointestinal tract (liraglutide, and tirzepatide). However, these drugs cause a small loss of BM and have such undesirable effects, such as constipation, urgency, paresthesia, nausea, and diarrhea [35]. Semaglutide and its analogues have recently appeared on the market, causing significant weight loss (up to 15%). Setmelanotide has also recently been registered for the treatment of severe genetic obesity [71].

The described somatic and mental states of the human body, also determined by body image, may be the reason for its acceptance [72] and can also be the expression of disapproval and deep impairments [69,73].

## 5. Limitations of the Presented Manuscript and Future Research Prospects

Among the factors limiting the current study is the relatively small group of people participating in it. At this point, we would like to point out that recruiting a larger number of participants for such a demanding experiment as 8 days of complete fasting and parallel physical exercise of maximum intensity is extremely difficult. Evidence of the above-mentioned difficulties is the lack of articles in the literature involving a larger number of people with such an extremely difficult research protocol. Various research protocols used by other authors, most often taking into account lighter forms of fasting and less intense physical exercise than those used in the presented study, make it difficult to compare the results obtained. We also believe that lifestyle, including the level of physical activity and the type of diet used, reflecting current somatic data and the degree of satisfaction with one’s body, may be factors limiting the current study. Another factor that limits drawing far-reaching conclusions based on this study is the participation of only men in it. However, it is known that women pay much more attention to the appearance of their own body than men. The age of the participants also seems to be a factor limiting this study because the body structure and its perception in different phases of ontogenesis are not the same. Various methods of correcting the structure and appearance of the body previously used may also affect the degree of its acceptance.

It was impossible to explain the above-mentioned limitations in the current study, but these doubts give impetus to conduct future multi-threaded experiments with various forms of fasting and physical exercise and with the participation of a larger number of volunteers of both sexes. It also seems that future research should be conducted by dividing participants into groups according to age, type of diet, or previous body corrections.

## 6. Conclusions

In conclusion, it should be said that the somatic aspects of the attractiveness of the human body are monitored not only by the interested party itself but also by the fields of cosmetology, aesthetic medicine, physical culture sciences, dietetics, and health sciences. An important, less objective but also more difficult to measure mental image of the human body described by psychology or sociology completes the image of the described object, and only then can one properly determine the degree of acceptance and image. The study we carried out, using simple almost cost-free tools, can have a wide public reception stimulating the initiation of changes in the unfavorable somatic conditions of the human body and also have improving mental effects on its imperfections.

## Figures and Tables

**Table 1:** Demographic and somatic variables (M ± SD; %) of volunteers before fasting.

	M ± SD	Minimum	Maximum		Higher	Secondary
Age	59.8 ± 12.37	37	77	Education	14 (93.33)	1 (6.66)
Height	178.133 ± 4.75	167.00	184.00	Marital status, number of people and %	YES 9 (60)	NO 6 (40)
Weight	88.240 ± 11.95	69.60	108.00	**Residence**	**Number of People**	**%**
BMI	27.800 ± 3.43	22.10	34.10	City > 200,000	9	60
Desired weight	78.923 ± 8.48	68.000	91.000	City of 50–200 thousand	4	26.67
				Village	2	13.33
	**Taking Dietary Supplements and Vitamins**					
	**D_3_**	**C**	**B_2_**	**B_6_**	**B_12_**	**E**
Number of people and %	9 (60)	7 (46.66)	2 (20)	5 (33.33)	2 (20)	1 (6.66)
	**Nutrition Rules Used**					
	**I Eat Everything I Feel Like Eating**	**I Strictly Plan My Diet**	**I Eat Only Healthy Food**	**I Follow a Vegetarian Diet**	**I Follow a** **Vegan Diet**	**I’m on a** **Keto Diet**
Number of people and %	7 (46.55)	0	5 (33.33)	4 (26.66)	5 (33.33)	0

**Table 2:** Comparisons of physiological indices of body composition before and after fasting.

Physiological Parametes	Before Fasting	Rank	Total Ranks	After Fasting	Rank	Total Ranks	T	Z	*p*
**Rest**									
BM [kg]	88.240 ± 11.95	–	–	82.967 ± 11.64	–	–	0.00	3.407	0.0001
BF [%]	23.560 ± 4.68	2.833	42.50	22.927 ± 4.78	2.967	44.50	33.00	1.533	0.125
BF [kg]	21.200 ± 6.78	2.333	35.00	19.273 ± 6.48	2.133	32.00	5.00	3.124	0.002
FFM [%]	76.465 ± 4.69	7.800	117.00	77.077 ± 4.78	7.933	119.00	33.00	1.533	0.125
FFM [kg]	67.047 ± 6.25	7.133	107.00	62.727 ± 5.83	6.733	101.00	0.00	3.408	0.001
TBW [%]	55.976 ± 3.44	5.867	88.00	56.425 ± 3.50	6.267	94.00	33.00	1.533	0.125
TBW [kg]	49.080 ± 4.56	5.200	78.00	45.920 ± 4.27	5.067	76.00	0.00	3.408	0.001
BMI [kg/m^2^]	27.800 ± 3.43	3.833	57.50	25.807 ± 3.29	3.900	58.50	0.00	3.408	0.001
β-HB [mmol/L]	0.142 ± 0.13	1.533	23.00	4.537 ± 1.63	3.733	56.00	0.00	3.408	0.001
C [ng/mL]	295.580 ± 92.10	1.533	23.00	468.633 ± 111.20	2.733	41.00	7.000	3.010	0.003
**Exercise**									
BM [kg]	88.227 ± 11.98	–	–	81.487 ± 11.23	–	–	14.00	2.613	0.009
BF [%]	22.757 ± 4.56	2.786	39.00	22.447 ± 4.68	9.000	126.00	0.00	3.296	0.001
BF [kg]	20.336 ± 6.68	2.357	33.00	18.820 ± 6.32	10.000	140.00	0.00	3.296	0.001
FFM [kg]	67.086 ± 6.25	8.000	112.00	63.047 ± 5.87	1.143	16.00	0.00	3.296	0.001
FFM [%]	76.734 ± 4.75	3.786	53.00	77.140 ± 4.66	9.600	144.00	33.00	1.533	0.125
TBW [kg]	49.100 ± 4.58	7.000	98.00	46.153 ± 4.29	6.000	84.00	0.00	3.296	0.001
TBW [%]	56.175 ± 3.48	2.071	29.00	56.355 ± 3.26	1.800	27.00	37.00	1.306	0.191
BMI [kg/m^2^]	27.543 ± 3.45	4.929	69.00	25.773 ± 3.29	3.786	53.00	0.00	3.296	0.001
β-HB [mmol/L]	0.114 ± 0.08	1.467	22.00	4.132 ± 1.56	3.267	49.00	0.00	3.407	0.001
C [ng/mL]	332.520 ± 111.62	2.067	31.00	529.620 ± 108.85	3.667	55.00	1.000	3.351	0.001

**Table 3:** Indications of body weight and subjective health assessment before and after fasting.

Variables	Before Fasting M ± SD	After Fasting M ± SD	Minimum	Maximum	T	Z	*p*
Body weight	88.240 ± 11.95	81.967 ± 11.645	69.600	108.00	0.00	2.934	0.003
Desired weight	78.923 ± 8.48	76.250 ± 7.81	64.000	101.80	1.00	1.069	0.285
Weight loss		6.273 ± 1.34	3.00	7.90			
Health assessment	7.533 ± 1.40	8.267 ± 0.96	4.00	10.00	3.00	2.310	0.020

**Table 4:** Level of satisfaction with selected body parts and parameters.

	Before Fasting			After Fasting						
Satisfaction	M ± SD	Rank	Total Ranks	M ± SD	Rank	Total Ranks	Difference	T	Z	*p*
Face	7.133 ± 1.68	6.200	93.00	7.200 ± 1.93	5.857	82.00	−0.067 ± 1.48	21.00	0.178	0.859
Chest	6.467 ± 2.41	5.567	83.50	7.200 ± 2.07	5.643	79.00	−0.733 ± 1.27	6.00	1.955	0.050
Arms	6.933 ± 1.98	5.967	89.50	7.467 ± 1.99	6.214	87.00	−0.533 ± 0.83	11.00	1.956	0.050
Waist	4.600 ± 2.58	3.100	46.50	6.000 ± 2.36	3.750	52.50	−1.400 ± 1.76	2.00	2.429	0.015
Belly	4.533 ± 2.82	2.867	43.00	5.467 ± 2.58	3.071	43.00	−0.933 ± 1.66	2.50	1.944	0.047
Thighs	6.714 ± 2.23	5.467	82.00	7.000 ± 2.11	4.929	69.00	−0.286 ± 0.82	16.50	1.121	0.262
Legs	6.133 ± 2.56	4.967	74.50	6.933 ± 2.01	5.393	75.50	−0.800 ± 2.67	25.50	1.398	0.162
Height	6.933 ± 2.37	6.467	97.00	7.267 ± 1.94	5.750	80.50	−0.333 ± 1.91	22.50	0.510	0.610
Weight	5.733 ± 2.84	4.400	66.00	6.533 ± 2.50	4.393	61.50	−0.800 ± 1.89	10.50	1.422	0.155

**Table 5:** Level of satisfaction with selected body parts and parameters—comparisons between variables measured before and after fasting.

Body Satisfaction Before Fasting		Body Satisfaction After Fasting	
Chi-Square and ANOVA (N = 15, df = 8) = 35.3410; *p* < 0.001; Coefficient of Concordance = 0.9451; r Mean Rank = 0.2441		Chi-Square and ANOVA (N = 15, df = 8) = 24.7873; *p* < 0.001; Coefficient of Compliance = 0.221; r Mean Rank = 0.1614	
Face	Waist, abdomen	Face	Waist, abdomen, mass
Chest	Waist *, belly *, height	Chest	Waist, abdomen, mass
Arms	Waist *, abdomen	Arms	Waist, abdomen, thighs
Waist	Face, abdomen, arms, thighs, legs, height	Waist	Face, chest, shoulders, abdomen, thighs, legs, height
Belly	Face, chest, arms, thighs, legs, height, weight	Belly	Face, chest, arms, waist, thighs, legs, height *, weight
Thighs	Waist *, abdomen	Thighs	Arms, waist, abdomen
Legs	Waist *, abdomen	Legs	Waist, abdomen
Height	Chest, waist *, abdomen	Height	Waist, abdomen, mass
Weight	Abdomen, height	Weight	Face, chest, abdomen, height

Note: Differences statistically significant for *p* < 0.05. * indicates *p* < 0.001.

**Table 6:** Body image dimensions and self-esteem and need for social approval.

	Before Fasting				After Fasting							
BIQ	M ± SD	Total	Rank	Total Ranks	M ± SD	Total	Rank	Total Ranks	Sten *	T	Z	*p*
Cognition-emotions	2.233 ± 12.82	35.733 ± 0.80	2.533	38.00	2.204 ± 0.72	35.267 ± 11.58	2.467	37.00	6	44.00	0.533	0.593
Behaviors	2.053 ± 3.86	10.266 ± 0.77	2.333	35.00	1.933 ± 0.81	9.667 ± 4.05	2.400	36.00	4	15.00	1.274	0.202
External criticism	1.711 ± 4.15	10.785 ± 0.64	1.133	17.00	1.611 ± 0.67	10.333 ± 3.99	1.133	17.00	5	28.00	0.862	0.388
Pretty-ugly stereotype	3.579 ± 7.70	46.533 ± 0.59	4.000	60.00	3.692 ± 0.68	48.000 ± 8.89	4.000	60.00	6	37.00	1.306	0.191
**BES**												
Physical attractiveness	3.697 ± 0.82	40.667 ± 9.06	1.667	25.00	3.764 ± 0.77	41.400 ± 8.46	1.933	29.00	6	36.50	0.629	0.529
Body strength	3.859 ± 0.87	34.733 ± 7.79	2.433	36.50	3.844 ± 0.82	34.600 ± 7.38	2.133	32.00	6	32.50	0.509	0.610
Physical condition	3.697 ± 0.83	48.06 ± 710.76	1.900	28.50	3.719 ± 0.83	48.353 ± 10.76	1.933	29.00	6	47.00	0.345	0.729
Self-Esteem Scale	3.213 ± 7.29	32.133 ± 0.73			3.433 ± 4.35	34.333 ± 0.44			7	29.50	1.444	0.149
Lie Scale (L Scale)-EPQ-R		7.667 ± 2.89				7.333 ± 3.92			5	45.00	0.471	0.638

Note: * The sten scale is used for the normalization of psychological tests, which makes it possible to compare specific scores of individuals or averages for groups to those of a normalization group. Thus, 1 sten is a very low score, 2–3 sten is a low score, 4 is a slightly reduced score, 5–6 is a normal score, 7 is a slightly increased score, 8–9 is a high score, and 10 is a very high score.

**Table 7:** Body image indices—comparisons between variables measured before and after fasting using Friedman’s ANOVA and Kendall’s Chi squared analysis.

BIQ—Before Fasting		BIQ—After Fasting	
ANOVA (N = 15, df = 3) = 7.8243, *p* = 0.00001; Coefficient of Concordance = 0.8405; r Mean Rank = 0.8291		ANOVA (N = 15, df = 2) = 0.4285, *p* = 0.807; Coefficient of Concordance = 0.01429; r Mean Rank = −0.0561	
Cognition-emotions (CE)	EC/PUS *	Cognition-emotions (CE)	EC/PUS *
Behaviors (B)	EC */PUS *	Behaviors (B)	EC */PUS *
External criticism (EC)	CE/B */PUS *	External criticism (EC)	CE/B */PUS *
Pretty-ugly stereotype (PUS)	CE */B */EC *	Pretty-ugly stereotype (PUS)	CE */B */EC *
**BES—Before Fasting**		**BES—After Fasting**	
**ANOVA (N = 15, df = 2) = 5.1481, *p* = 0.0762;** **Coefficient of Compliance = 0.1716,** **r Mean Rank = 0.1124**		**ANOVA (N = 15, df = 3) = 37.1600, *p* = 0.00001;** **Coefficient of Concordance = 0.8257, r Mean Rank = 0.8133**	
Physical attractiveness (PA)	BS	Physical attractiveness (PA)	Lack of differences
Body strength (BS)	Lack of differences	Body strength (BS)	Lack of differences
Physical condition (PC)	Lack of differences	Physical condition (PC)	Lack of differences

Note: Differences statistically significant for *p* < 0.05. * indicates *p* < 0.001. The symbols used in the table correspond to the subsequent scales of BIQ and BES, respectively: CE—Cognition-emotions, B—Behaviors, EC—External criticism, PUS—Pretty-ugly stereotype, PA—Physical attractiveness, BS—Body strength, PC—Physical condition.

**Table 8:** Use and evaluation of the effectiveness of the weight control.

Using Different Ways to Control Body Weight	Before Fasting	Rank	Total Ranks	After Fasting	Rank	Total Ranks	T	Z	*p*
a. Limiting the amount of food	5.571 ± 2.24	6.2143	87.00	6.067 ± 2.43	6.3667	95.50	16.50	1.121	0.262
b. Elimination of sugar	6.800 ± 2.57	6.8929	96.50	7.400 ± 1.68	6.9667	104.50	13.00	1.478	0.139
c. Eliminating fatty foods	6.267 ± 2.60	6.4286	90.00	6.333 ± 2.35	6.3333	95.00	20.50	0.237	0.813
d. Eliminating meat	6.267 ± 3.06	6.5000	91.00	6.533 ± 2.61	6.2667	94.00	12.50	0.770	0.441
e. Restrictive diet	4.000 ± 2.65	5.2143	73.00	4.867 ± 2.88	4.9333	74.00	13.50	1.427	0.154
f. Fasting	6.000 ± 2.98	6.4286	90.00	5.867 ± 2.64	6.2000	93.00	25.50	0.204	0.838
g. Appetite suppressants	1.333 ± 1.29	2.3214	32.50	2.000 ± 2.48	2.8333	42.50	1.00	1.069	0.285
h. Laxatives	1.400 ± 0.00	2.1071	29.50	1.667 ± 1.55	2.4333	36.50	12.00	0.338	0.735
i. Provoking vomiting	1.000 ± 0.63	2.8929	40.50	1.400 ± 1.40	2.6667	40.00	-	-	-
**Evaluation of the Effectiveness of** **Different Weight Control Methods**	**Before** **Fasting**	**Rank**	**Total Ranks**	**After Fasting**	**Rank**	**Total Ranks**	**T**	**Z**	***p***
a. Limiting the amount of food	8.000 ± 1.13	6.9667	104.50	7.533 ± 2.00	7.0357	98.50	31.500	0.588	0.556
b. Elimination of sugar	7.929 ± 1.00	7.1333	107.00	7.643 ± 1.45	7.0357	98.50	11.500	0.910	0.362
c. Eliminating fatty foods	6.500 ± 1.91	5.6000	84.00	7.643 ± 1.45	5.2857	74.00	33.000	0.873	0.382
d. Eliminating meat	6.067 ± 2.37	5.1000	76.50	5.933 ± 2.34	4.9643	69.50	30.000	0.266	0.789
e. Restrictive diet	5.800 ± 2.73	4.9333	74.00	5.800 ± 2.14	4.7857	67.00	31.000	0.177	0.858
f. Fasting	8.200 ± 1.42	7.2667	109.00	7.600 ± 2.03	7.0714	99.00	6.000	0.943	0.345
g. Appetite suppressants	3.733 ± 1.71	2.8000	42.00	3.733 ± 1.87	3.0714	43.00	5.000	0.000	1.000
h. Use of laxatives	3.800 ± 2.27	2.2000	33.00	3.800 ± 1.78	2.7143	38.00	10.500	0.000	1.000
i. Provoking vomiting	2.867 ± 2.07	3.0000	45.00	3.533 ± 1.88	3.0357	42.50	2.000	1.483	0.138

**Table 9:** Use of different weight control methods and evaluation of their effectiveness-comparisons between variables measured before and after fasting with Friedman’s ANOVA and Kendall’s Chi squared analysis.

	Use of		Evaluation of Effectiveness	
Weight Control Methods	Before Fasting	After Fasting	Before Fasting	After Fasting
Limiting the amount of food (LAF)	AS/UL/PV	RD/AS/UL/PV	EM/AS */UL */PV *	EFF/EM/RD/AS */PV */UL *
Elimination of sugar (ES)	RD/F/AS/h	RD/AS/UL/PV	EFF/EM/RD/g	RD/AS */UL */PV *
Eliminating fatty foods (EFF)	AS/UL/PV	RD/ASUL/PV	ES/AS/UL/PV	AS/UL/PV
Eliminating meat (EM)	EM/AS/UL/PV	AS/UL/PV *	LAF/ES/F/AS/UL/PV	LAF/PV
Restrictive diet (RD)	AS/UL/PV	F/AS/UL/PV	ES/F/PV	F/UL/PV
Fasting (F)	ES/AS/UL/PV	RD/AS/UL/PV	F/AS */UL */PV *	AS/UL */PV *
Appetite suppressants (AS)	LAF/ES/EFF/EM/RD/F	LAF/ES/EFF/EM/RD/F	LAF/ES/EFF/EM /F */PV	LAF/ES */EFF/F
Use of laxatives (UL)	LAF/ES/EFF/EM/RD/F	LAF/ES/EFF/EM/RD/F	LAF/EFF/EM/F	LAF */ES */EFF/RD/F
Provoking vomiting (PV)	LAF/EFF/EM/RD/F	LAF/ES/EFF/EM/RD/F	LAF/EFF/EM/RD/F */AS	LAF */ES */EFF/EM/RD/F
	ANOVA (N = 15. df = 8) = 65.16402, *p* = 0.00001 Coefficient of compliance = 0.5818, r mean rank = 0.5496	ANOVA (N = 15. df = 8) = 60.9051, *p* =0.00001; concordance coefficient = 0.5075, r mean rank = 0.4723	ANOVA (N = 15, df = 8) = 69.8352, *p* = 0.00001; coefficient of compliance = 0.5819, r mean rank = 0.5521	ANOVA (N = 15, df = 8) = 5.4217, *p* =0.00001; coefficient of compliance = 0.4859, r mean rank = 0.4463

Note: Differences statistically significant for *p* < 0.05. * indicates *p* < 0.001. The symbols used in the table correspond to the subsequent weight control methods as follows: LAF—limiting the amount of food; ES—elimination of sugar; EFF—eliminating fatty foods; ED—eliminating meat; RD—restrictive diet; F—fasting; AS—appetite suppressants; UL—use of laxatives; PV—provoking vomiting.

**Table 10:** Correlations between satisfaction with appearance and body parameters and selected physiological parameters measured at rest and post-exercise.

Rest	Before Fasting								After Fasting					
	BF [%]	BF [kg]	FFM [%]	FFM [kg]	TBW [%]	TBW [kg]	BMI [kg/m^2^]	BF [%]	BF [kg]	FFM [%]	FFM [kg]	TBW [%]	TBW [kg]	BMI [kg/m^2^]
Face	0.039	0.024	−0.042	−0.103	−0.042	−0.103	−0.143	−0.305	−0.329	0.305	−0.310	0.30	−0.310	−0.363
Chest	0.213	0.136	−0.190	−0.297	−0.190	−0.297	−0.116	−0.087	−0.199	0.087	−0.401	0.09	−0.401	−0.325
Arms	0.072	0.062	−0.060	−0.236	−0.060	−0.236	−0.007	−0.224	−0.340	0.224	−0.494	0.22	−0.494	−0.322
Waist	0.176	0.099	−0.155	−0.321	−0.155	−0.321	−0.200	−0.126	−0.146	0.126	−0.150	0.13	−0.150	−0.322
Belly	0.202	0.136	−0.182	−0.236	−0.182	−0.236	−0.173	−0.153	−0.195	0.153	−0.209	0.15	−0.209	−0.355
Thighs	−0.114	−0.093	0.125	−0.205	0.125	−0.205	−0.134	0.051	−0.007	−0.051	−0.124	−0.05	−0.124	−0.136
Legs	0.378	0.383	−0.372	0.116	−0.372	0.116	0.258	−0.087	−0.057	0.087	−0.009	0.09	−0.009	−0.066
Height	0.309	0.325	−0.307	0.070	−0.307	0.070	0.079	−0.295	−0.331	0.295	−0.346	0.30	−0.346	−0.471
Weight	0.237	0.132	−0.214	−0.373	−0.214	−0.373	−0.243	−0.430	−0.486	0.430	−0.570	0.43	−0.570	−0.652
**Exercise**	**Before Fasting**								**After Fasting**					
	**BF [%]**	**BF [kg]**	**FFM [%]**	**FFM [kg]**	**TBW [%]**	**TBW [kg]**	**BMI [kg/m^2^]**	**BF [%]**	**BF [kg]**	**FFM [%]**	**FFM [kg]**	**TBW [%]**	**TBW [kg]**	**BMI [kg/m^2^]**
Face	0.029	0.056	0.129	−0.070	0.129	−0.070	−0.117	−0.335	−0.333	0.274	−0.335	0.183	−0.335	−0.354
Chest	0.101	0.087	0.095	−0.327	0.095	−0.327	−0.143	−0.117	−0.218	0.195	−0.428	0.202	−0.428	−0.316
Arms	−0.020	0.043	0.307	−0.251	0.307	−0.251	−0.005	−0.252	−0.340	0.381	−0.501	0.409	−0.501	−0.312
Waist	0.132	0.075	−0.051	−0.291	−0.051	−0.291	−0.188	−0.155	−0.139	−0.072	−0.177	−0.090	−0.172	−0.311
Belly	0.173	0.132	−0.014	−0.208	−0.014	−0.208	−0.163	−0.186	−0.197	0.019	−0.242	−0.005	−0.237	−0.347
Thighs	−0.190	−0.087	−0.046	−0.205	−0.046	−0.205	−0.136	0.032	−0.018	0.163	−0.145	0.161	−0.135	−0.116
Legs	0.269	0.337	−0.062	0.080	−0.062	0.080	0.212	−0.104	−0.081	0.197	−0.034	0.147	−0.026	−0.049
Height	0.301	0.369	0.032	0.136	0.032	0.136	0.111	−0.325	−0.293	0.173	−0.373	0.132	−0.373	−0.462
Weight	0.148	0.022	0.182	−0.407	0.182	−0.407	−0.278	−0.452	−0.450	0.274	−0.597	0.281	−0.591	−0.646

Correlations statistically significant at *p* < 0.05.

**Table 11:** Correlations between body image dimensions and selected physiological parameters measured at rest and after exercise.

	Before Fasting							After Fasting						
	Cognition-Emotion	Behaviors	External Criticism	Pretty-Ugly Stereotype	Physical Attractiveness	Body Strength	Physical Condition	Cognition-Emotion	Behaviors	External Criticism	Pretty-Ugly Stereotype	Physical Attractiveness	Body Strength	Physical Condition
**Rest**														
BF [%]	0.261	−0.137	0.284	−0.146	−0.315	−0.096	−0.103	0.215	0.000	0.133	−0.084	−0.021	0.241	−0.132
BF [kg]	0.304	−0.079	0.320	−0.197	−0.343	−0.100	−0.135	0.262	−0.054	0.191	−0.027	−0.011	0.189	−0.082
FFM [%]	−0.274	0.115	−0.287	0.148	0.334	0.118	0.128	−0.215	0.000	−0.133	0.084	0.021	−0.241	0.132
FFM [kg]	0.444	0.244	0.403	−0.207	−0.472	−0.172	−0.234	0.330	0.022	0.250	−0.122	−0.086	0.036	−0.114
TBW [%]	−0.274	0.115	−0.287	0.148	0.334	0.118	0.128	−0.215	0.000	−0.133	0.084	0.021	−0.241	0.132
TBW [kg]	0.444	0.244	0.403	−0.207	−0.472	−0.172	−0.234	0.330	0.022	0.250	−0.122	−0.086	0.036	−0.114
BMI [kg/m^2^]	0.639	0.189	0.643	0.016	−0.492	−0.305	−0.255	0.376	0.029	0.211	0.118	−0.102	0.075	−0.143
Exercise														
BF [%]	0.289	−0.147	0.302	−0.128	−0.433	−0.234	−0.241	0.254	0.011	0.179	−0.070	−0.043	0.205	−0.154
BF [kg]	0.295	−0.142	0.326	−0.179	−0.401	−0.176	−0.223	0.244	0.004	0.202	−0.081	−0.016	0.169	−0.141
FFM [%]	−0.825	−0.159	0.004	−0.231	0.138	−0.084	−0.016	0.057	−0.022	−0.011	0.145	−0.313	−0.456	0.047
FFM [kg]	0.420	0.168	0.418	−0.231	−0.523	−0.270	−0.333	0.333	0.065	0.251	−0.061	−0.097	0.041	−0.143
TBW [%]	−0.159	0.004	−0.231	0.326	0.138	−0.084	−0.016	0.057	−0.022	−0.011	0.145	−0.313	−0.456	0.047
TBW [kg]	0.420	0.168	0.418	−0.231	−0.523	−0.270	−0.333	0.344	0.058	0.273	−0.077	−0.106	0.025	−0.141
BMI [kg/m^2^]	0.615	0.115	0.625	0.027	−0.514	−0.366	−0.325	0.371	0.011	0.212	0.104	−0.088	0.083	−0.130

Correlations statistically significant at *p* < 0.05.

**Table 12:** Correlations between body image indices and satisfaction with appearance and body parameters.

	Before Fasting							After Fasting						
	BIQ				BES			BIQ				BES		
Body Parts and Parameters	Cognition-Emotion	Behaviors	External Criticism	Pretty-Ugly Stereotype	Physical Attractiveness	Body Strength	Physical Condition	Cognition-Emotion	Behaviors	External Criticism	Pretty-Ugly Stereotype	Physical Attractiveness	Body Strength	Physical Condition
Face	−0.661	−0.441	−0.747	−0.680	0.406	0.333	0.301	−0.554	−0.268	−0.553	−0.506	0.638	0.244	0.550
Chest	−0.558	−0.563	−0.342	−0.333	0.633	0.749	0.679	−0.571	−0.643	−0.417	−0.456	0.469	0.594	0.472
Arms	−0.186	−0.143	−0.108	−0.209	0.703	0.529	0.527	−0.298	−0.316	−0.288	−0.359	0.433	0.286	0.384
Waist	−0.645	−0.595	−0.483	−0.572	0.508	0.595	0.641	−0.403	−0.314	−0.318	−0.494	0.384	0.418	0.714
Belly	−0.704	−0.691	−0.559	−0.589	0.471	0.635	0.638	−0.516	−0.450	−0.419	−0.524	0.375	0.484	0.673
Thighs	−0.385	−0.309	−0.347	−0.419	0.790	0.686	0.684	−0.410	−0.684	−0.207	−0.566	0.483	0.579	0.403
Legs	−0.270	−0.219	−0.155	−0.440	0.415	0.457	0.336	−0.152	−0.494	−0.197	−0.266	0.537	0.549	0.568
Height	−0.569	−0.505	−0.545	−0.655	0.318	0.396	0.248	−0.856	−0.484	−0.657	−0.515	0.424	0.406	0.308
Weight	−0.485	−0.373	−0.330	−0.378	0.408	0.474	0.498	−0.601	−0.301	−0.346	−0.417	0.358	0.284	0.355

Correlations statistically significant at *p* < 0.05.

## Data Availability

The datasets generated and analyzed during the study are not publicly available due to data protection reasons and proprietary rights, but are available from the correspondence author upon reasonable request.
